# Friction and Wear Characteristics of 18Ni(300) Maraging Steel under High-Speed Dry Sliding Conditions

**DOI:** 10.3390/ma13071485

**Published:** 2020-03-25

**Authors:** Kun Sun, Weixiang Peng, Binghui Wei, Longlong Yang, Liang Fang

**Affiliations:** State Key Laboratory for Mechanical Behavior of Materials, Xi’an Jiaotong University, Xi’an 710049, China; thunder0@stu.xjtu.edu.cn (W.P.); wfzgb8210@stu.xjtu.edu.cn (B.W.); yanglonglong@stu.xjtu.edu.cn (L.Y.); fangl@xjtu.edu.cn (L.F.)

**Keywords:** 18Ni(300) maraging steel, friction coefficient, oxides, wear mechanism

## Abstract

18Ni(300) maraging steel, which has exceptional strength and toughness, is used in the field of aviation and aerospace. In this paper, using a high-speed tribo-tester, tribological behaviors of 18Ni(300) maraging steel were investigated under high-speed dry sliding conditions. Morphology of the worn surfaces and the debris was analyzed by scanning electron microscope, and the oxides of worn surfaces caused by friction heat were detected by X-ray diffraction. The experiment results reveal that the friction coefficient of frictional pairs declines with increasing load and speed. With the speed and load increasing, oxides of the worn surfaces of 18Ni(300) maraging steel change from FeO to Fe_3_O_4_ and the wear mechanism converts from adhesive wear into severe oxidative or extrusion wear.

## 1. Introduction

18Ni(300) maraging steel is a kind of high-alloy maraging steel, depending on 9% cobalt as its strengthening agent, and has ultra-high strength, high toughness, good machinability, and weldability. It is widely used in pressure vessels, aircraft components, rocket motor cases, hot-work dies, and high-speed rocket-propelled sleds. Therefore, much research about the steel has been carried out, such as investigation of its microstructure and mechanical properties, strengthening mechanism, the effects of solid solution, and aging treatment and so on. Li and Yin [1] examined the effect of age time and temperature on the evolution of reverted austenite in 18Ni(350) maraging steel. Pardal et al. [2] investigated the austenite and martensite phases of 18Ni(300) maraging steel at different temperatures and periods of time and revealed that the martensite parameter increased with the aging time above 1 h at 560–600 °C. Shamantha et al. [3] studied the microstructure of 18Ni(250) maraging steel changes during welding and subsequent heat treatment. Shvetsov et al. [4] studied structure, mechanical properties, and crack resistance of 18Ni maraging steel after tempering treatment and found the range of tempering temperatures is a key to ensure the combination of properties required. With the progress of technology, 18Ni(300) maraging steel can also be used as the tribo-pair material of sleds under high speed, large loads, or other severe sliding conditions. Burton and Brockman [5] investigated the dependence of the appropriate friction coefficient of 18Ni(300) maraging steel upon load and velocity using simulation software (ABAQUS). Hale et al. [6] predicted the wear rate of 18Ni(300) maraging steel during a high-speed dry sliding process using ABAQUS with a surface collision numerical model. Yin et al. [7] investigated the wear-resistance performance of 18Ni(300) maraging steel fabricated by selective laser melting technology using a pin-on-disc tribometer at room temperature and a speed of 10 mm/s. They found that aging treatment could improve the tribological properties of the maraging steel, and the steel had the best wear-resistance performance due to the improved hardness and strength under optimal aging conditions (490 °C for 3 h). The wear mechanism of maraging steel changed from abrasion wear in the as-fabricated state to adhesion tribofilm in the optimally aged state.

However, friction and wear behaviors of 18Ni(300) maraging steel under high-speed dry sliding conditions (>10 m/s) have not been reported in detail. Thus, the aim of this paper is to study the wear mechanisms and behavior transitions of 18Ni(300) maraging steel under high-speed dry sliding conditions.

## 2. Experimental Procedure

**Figure 1:**
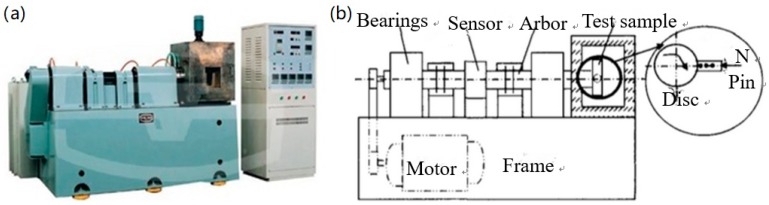
(**a**) The pin-on-disk tester; (**b**) a schematic illustration of the tester.

The dry sliding wear tests were conducted under atmospheric conditions on a pin-on-disk high-speed tribo-tester (MMs-1G type, Shijing Machine Factory, Jinan, China). A schematic diagram of the wear tester used in this study is shown in Figure 1. The disk is fixed to the rotating shaft in the tester. The pin specimen is pressed against the bottom surface of the disk under a constant vertical load. 18Ni(300) maraging steel, manufactured by double vacuum melting, was employed to fabricate the pin specimens. Disk specimens were fabricated using commercial 100Cr6 steel. Chemical compositions of pin and disk specimens are shown in Table 1.

**Figure 2:**
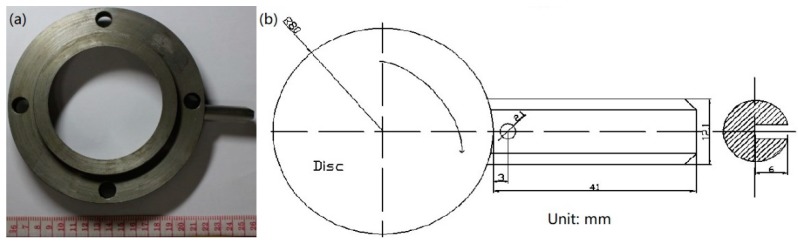
(**a**) Pin and disk specimens; (**b**) shape and size of specimens.

**Figure 3:**
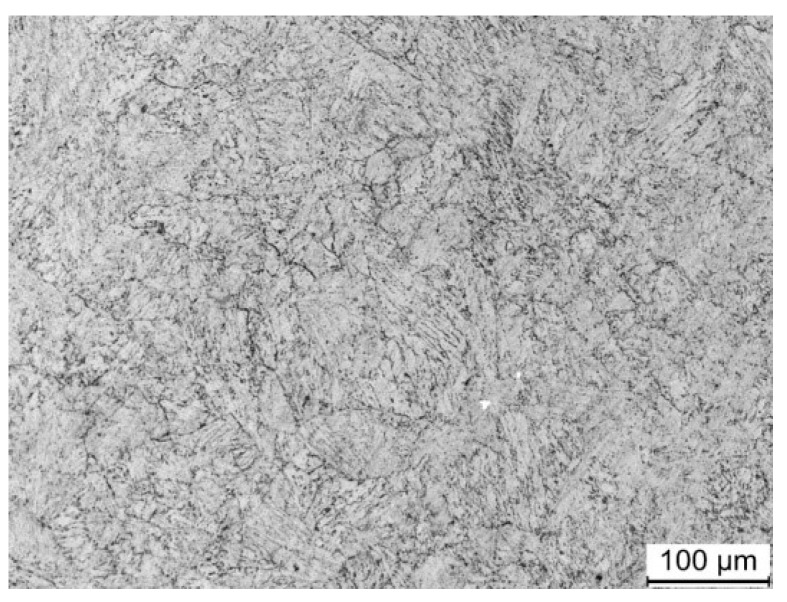
SEM image of 18Ni(300) maraging steel after heat treatment.

The pin and disk specimens are illustrated in Figure 2 and have the dimensions of ϕ12 mm×41 mm and φ160 mm×30 mm, respectively. The 18Ni(300) maraging steel was solution-treated at 820 °C for 60 min, then aged at 480 °C for 6 h and cooled in air. After heat treatment, the microstructure of the pin specimens was a martensite structure with a dispersedly distributed metal compound strengthening phase, as shown in Figure 3. The yield stress and hardness of the pin specimens were 1930 MPa and HRC53, respectively. After quenching and tempering, the hardness of the 100Cr6 steel specimens was 58 HRC.

Wear tests were carried out under sliding speeds in the range of 30–60 m/s and applied loads in the range of 100–300 N (contact pressures were in the range from 0.65 to 1.95 MPa, correspondingly) at a sliding distance of 16 km. The initial roughness of the sliding surfaces of pin and disk specimens was about *Ra* = 0.5 μm. Three tests for each experimental point were carried out, and the average value of the three test results was calculated as the experiment data point. All the data of wear loss only were measured from pin specimens because the main aim of the present work is to study the friction and wear characteristics of 18Ni(300) maraging steel under high-speed dry sliding conditions. Before and after testing, pin specimens were cleaned, dried, and then weighed for wear loss with a balance with an accuracy of 0.01 mg. The friction coefficient and contact temperature of pin specimens were also recorded during the tribo-test process. For the temperature measurements, a NiCr-NiAl type thermocouple was placed in the hole (with a diameter of 2.1 mm) at a distance of 3 mm from the contact surface of pin specimens. The average surface temperature was then obtained by considering a linear heat flow and using the approach developed by Zhang and Alpas [8].

The structure, morphology, and composition of the worn surfaces of pin specimens and wear debris were analyzed using a JSM-7001F type (JEOL Ltd., Tokyo, Japan) scanning electron microscope (SEM) equipped with energy-dispersive spectrum (EDS) and X-ray diffractometer (XRD), respectively.

## 3. Results and Discussions

### 3.1. Friction and Wear Properties

**Figure 4:**
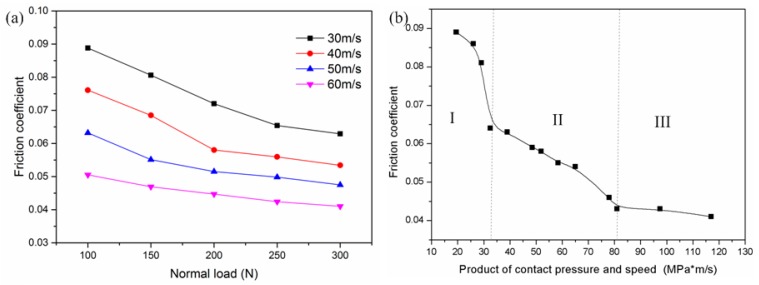
Friction coefficients: (**a**) at various loads vs. (**b**) the product of contact pressure and speed.

The friction coefficients of 18Ni(300) maraging steel under various load and speed conditions are shown in Figure 4a. It can be found that the friction coefficient decreased with the speed and load increasing. The main reason for the decrease can be attributed to high values of normal loads combined with high slip speeds, which generate substantial heat at the contact interface [9,10]. This friction heat softens the material at contact junctions and reduces their shear strength resisting plastic deformation. Thus, the frictional resistance and friction coefficient are decreased in turn. The friction coefficients as a function of the product of contact pressure and sliding speed are plotted in Figure 4b. It can be found that the friction coefficient steadily decreased with the increasing product of speed and contact pressure. The variation of the friction coefficient may be divided into three regions according to the descending rate of the friction coefficient shown in Figure 4b. In region I, the friction coefficient abruptly drops when the product is less than 33 MPa·m/s. In region II, the friction coefficient normally falls when production is from 33 to 81 MPa·m/s. When the product is larger than 81 MPa·m/s, the variation of the friction coefficient is very smooth and belongs to region III;. These changes in the variation rate of the friction coefficient reveal that there may be a transformation of the wear mechanism of 18Ni(300) maraging steel during the high-speed sliding process. This is further investigated in the next section.

**Figure 5:**
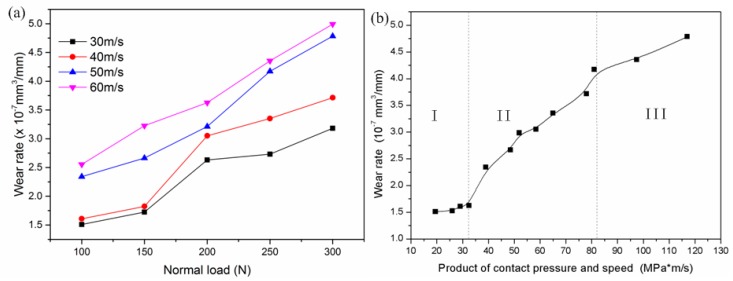
Wear rate: (**a**) at various loads vs. (**b**) the product of contact pressure and speed.

Wear rates of pin specimens were calculated according to following formula:

(1) $$W_{s}=\frac{\Delta V}{d}$$

where ΔV is the wear loss volume and *d* the sliding distance. The curves of wear rate vs. load at various sliding speeds are displayed in Figure 5a. It can be observed from Figure 5a that the wear rate generally increased with the increasing of loads and sliding speeds, and curves of wear rate at 30 and 40 m/s were in similar form. When the sliding speed was lower than 50 m/s, the wear rate of 18Ni(300) maraging steel slowly increased under low loads 100 and 150 N, then rapidly increased as the load was elevated from 150 to 200 N. It is clear that a transition of mild to severe wear occurred with the increasing of load. At the same time, it was also found that the wear rate linearly increased with the increasing of load at 50 and 60 m/s. This reveals that at higher sliding speed (>50 m/s), the wear mechanism of 18Ni(300) maraging steel is stable, and wear severe. The wear rate as a function of the product of contact pressure and speed is shown in Figure 5b. It is similar to the variation of the friction coefficient and can be divided into three regions, correspondingly. When the product is less than 33 MPa·m/s in region I, the wear rate is smaller, and increasing slowly. In region II, the wear rate normally elevates when the product is rising from 33 to 81 MPa·m/s. When the product is larger than 81 MPa·m/s, the wear rate slowly increases again. These changes of wear rate also reveal the dominant wear mechanism transferred from one region to the next region.

**Figure 6:**
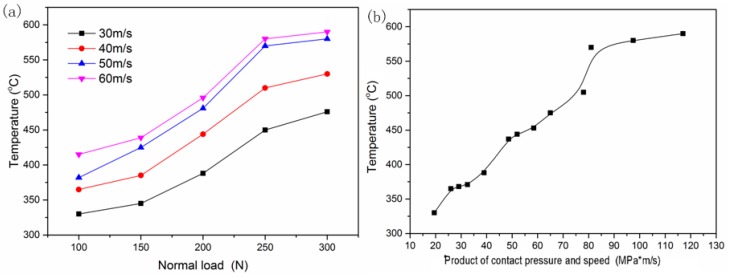
The temperature of contact surface: (**a**) at various loads vs. (**b**) the product of contact pressure and speed.

The nominal temperatures of the worn surface at various rubbing conditions are shown in Figure 6. It can be detected from Figure 6a that the effects of sliding speed and load on the temperature are similar and obvious. The nominal temperature of the contact area was about 330 °C at speed 30 m/s and load 100 N and then reached a critical 590 °C at speed 60 m/s and load 300 N. The contact temperature was high enough to cause local junction region oxidation; the oxidation products of worn surfaces are investigated in detail in the following section. It can also be found that the contact temperature was a function of the product of contact pressure and speed, shown in Figure 6b. With the product increasing, the contact temperature rose. However, the increasing rate was obviously slower when the product was larger than 81 MPa·m/s.

### 3.2. Phase and Morphology of Worn Surfaces and Debris

**Figure 7:**
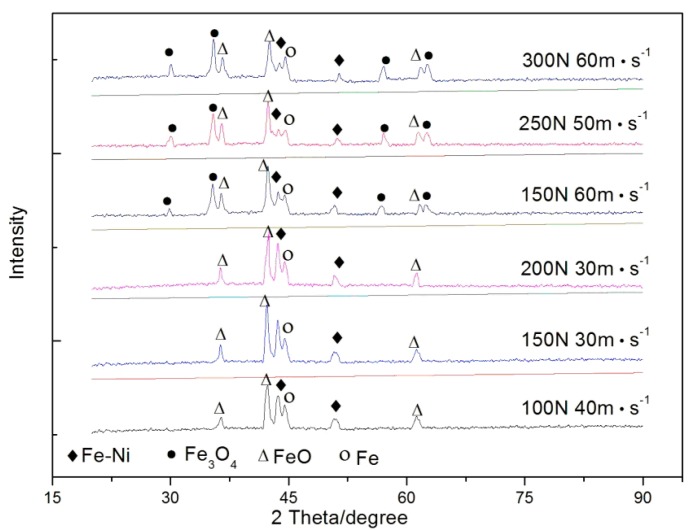
XRD patterns of worn surfaces of 18Ni(300) maraging steel.

XRD patterns of pin specimens under different sliding conditions are shown in Figure 7. At 100 N and 40 m/s (namely 26 MPa·m/s), ferrous oxides (FeO) were formed on worn surfaces. With increasing load and speed, the peak height of FeO approached or surpassed that of steel substrate. This means that massive FeO formed on worn surfaces of pin specimens. When sliding speed reached 60 m/s and load was 150 N (namely 58.5 MPa·m/s), a new type of oxide, spinel oxide (Fe_3_O_4_), also appeared on worn surfaces. However, FeO was still identified as a predominant oxide at this time. With further raising of the load, the amount of Fe_3_O_4_ increased substantially and significantly surpassed that of FeO. According to the result of XRD analyses, it is reasonable to deduce the predominant wear mechanism of 18Ni(300) maraging steel under high-speed dry sliding conditions may be oxidation wear, because a great amount of tribo-oxides appeared on worn surfaces of the steel [11].

**Figure 8:**
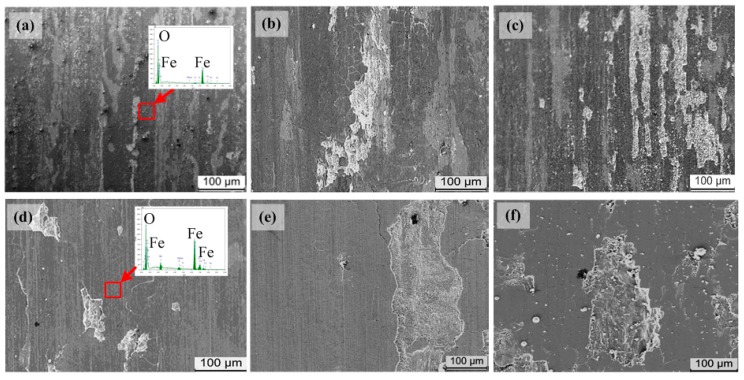
Morphologies of worn surfaces of 18Ni(300) maraging steel under (**a**) 100 N and 40 m/s, (**b**) 150 N and 30 m/s, (**c**) 200 N and 30 m/s, (**d**) 150 N and 60 m/s, (**e**) 200 N and 50 m/s, and (**f**) 300 N and 60 m/s.

SEM images of the worn surface of pin specimens under different load and speed conditions are shown in Figure 8. It can be found that adhesion occurred on worn surfaces, from Figure 8a. At the same time, composition of worn surfaces in red squares was analyzed using EDS. According to the EDS results, it was found that some regions of the worn surfaces were oxidized at 100 N and 40 m/s (namely, 26 MPa·m/s). Combining the XRD results (seen in Figure 7), the predominant oxide in these regions was detected as FeO. With the increase of load and speed as shown in Figure 8b,c, the adhesive wear changed from mild to severe. Some micro cracks appeared in the tribo-oxide films, and part of the films spalled off and delaminated from worn surfaces [11,12]. These phenomena reveal that a transition of wear mechanism of 18Ni(300) maraging steel occurs at 150 N and 30 m/s (namely, 33 MPa·m/s). On the other hand, it is interesting to find that the worn surfaces were very smooth at higher load and sliding speed (Figure 8d–f). Moreover, the predominant oxide transfers from FeO to Fe_3_O_4,_ according to XRD results shown in Figure 7. Simultaneously, it could be observed that delaminated regions became larger and deeper on the worn surfaces of pin specimens, and severe oxidative wear prevailed [11,12]. Under severe wear conditions, the increasing of speed and load caused a massive amount of frictional heat, which raised the worn surface temperature and caused a greater temperature gradient within the rubbing metal. The result was huge thermal stress in the subsurface layer of rubbing pairs. At interaction of the thermal stress and contact shear stress, more micro fatigue cracks were initiated and grew in the substrate of the rubbing metals, and between the oxide film and the substrate. These enhanced the delaminating of oxide films. Consequently, the wear rate increased with the product of contact pressure and sliding speed increasing.

According the above XRD and SEM results (Figure 7 and Figure 8), we can see that the main wear mechanism of 18Ni(300) maraging steel is mild adhesive wear when the oxide of friction and wear surface is FeO. With increasing speed and load, mild adhesive wear will transform to oxidative wear as the predominant oxide changes from FeO to Fe_3_O_4_. The Fe_3_O_4_ oxide films densely covered worn surfaces of pin and disk, and prevented metal–metal direct contact and adhesion [13,14], resulting from the decrease of the friction coefficient. However, with increasing sliding speed and load, the contact temperature between the pin-disk rubbing pairs rose, which caused larger plastic deformation in Fe_3_O_4_ oxide films and the subsurface of the matrix. As a result, the protective oxide film was broken into many small pieces by surface cracks and spalled off from the worn surfaces. Simultaneously, the shedding oxide debris scratched the rubbing surface as an abrasive, which further worsened the friction and wear properties of materials.

**Figure 9:**
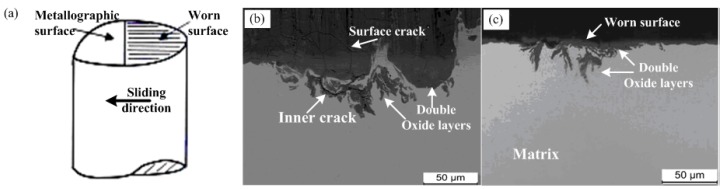
Section morphologies of worn surface of 18Ni(300) maraging steel under 300 N and 60 m/s: (**a**) schematic diagram of the clip section; (**b**) clip section morphology; and (**c**) cross section morphology.

Morphologies of clip sections and cross sections of worn pin specimens at 300 N and 60 m/s (namely, 117 MPa·m/s) are shown in Figure 9b,c. A schematic diagram of the clip section is present in Figure 9a, and the clip plane is inclined to the worn surface about 20°. It can be found from Figure 9b that some cracks formed in oxide films of worn surfaces, then propagated within the matrix. Eventually, these cracks in the subsurface led to a massive oxide film breaking into lots of small pieces and peeling off. The thickness and shape of the oxide layer can be also observed and measured from the morphology of cross sections. The thickness of the oxide layer is about 40 μm in Figure 9c. Meanwhile, double-layer tribo-oxides were formed in the cross section of worn surfaces under larger load and higher speed conditions, such as 300 N and 60 m/s. The appearance of double-layer oxides should be attributed to two reasons [15,16]: One reason is that there was severe plastic deformation and high thermal softening in the contact region under larger load and higher speed conditions, which caused the oxide films on worn surfaces to break and mechanically mix into the subsurface layer of the matrix; the other reason is that the deformation and softening also caused lots of micro cracks to form in the subsurface of the matrix and then expand to the worn surfaces, which accelerated oxygen penetrating into the inner matrix. Combining the result of wear rate tests (Figure 5), it can be found that the wear rate of 18Ni(300) maraging steel is very high when multi-layer tribo-oxides film form in matrix, and about thrice that at low load and speed (e.g., 100 N, 30 m/s). Thus, the wear mechanism in this situation should be distinguished as severe oxidative and plastic wear.

**Figure 10:**
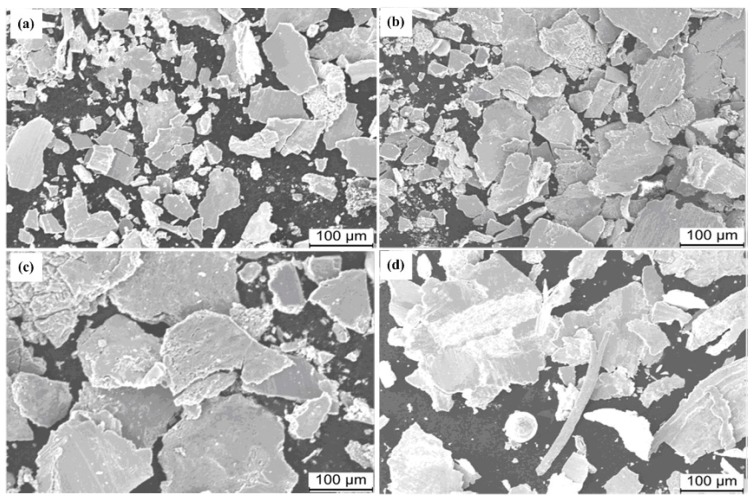
Morphologies of worn debris of 18Ni(300) maraging steel under (**a**) 100 N and 30 m/s, (**b**) 150 N and 40 m/s, (**c**) 250 N and 50 m/s, and (**d**) 300 N and 60 m/s.

SEM micrographs of debris of pin specimens under different speed and load conditions are presented in Figure 10. When sliding speed and load were relatively small, the debris was mainly little pieces of flake and chip, shown in Figure 10a,b. With increasing sliding speed and load, the shape of debris changed from little flakes to massive blocks, shown in Figure 10c. These massive blocks of debris were thick and irregular, which is the result of severe plastic deformation. With the sliding speed increasing, some spherical debris appeared, as shown in Figure 10d. It was the result of melted material extruding from the worn surface, and spontaneously solidifying into spherical shape during the cooling process in order to reduce the surface energy. When fine particles are found in debris (Figure 10d), this means that the wear transformed from adhesive and mild oxidative wear to severe oxidative and plastic wear.

### 3.3. Wear Mechanism Analysis

The transformation of the wear mechanism was closely related to contact temperature and friction heat in the worn surface layer, because friction heat has important influences on wear behavior and accelerates the formation of tribo-oxides in a rubbing system [17]. The heat mainly originated from plastic deformation of rubbing pairs and is determined by the product of friction force and sliding speed. The relationship of friction heat and load speed is described as follows:

(2) $$q=\frac{\mu F_{N}v}{A}$$

where μ is the friction coefficient, *F_N_* normal load, *v* sliding speed, and *A* contact area.

During the dry sliding test process, bare metal–metal contacted in some small areas and formed a junction when the pin was pushed against the disk specimen. Under a low load of 100 N and sliding speed of 30 m/s, the plastic deformation or friction heat, *q*, was small, and limited in those junction regions, where ferrous oxide (FeO) films may be formed. The oxide films were thin (about 8 μm) and isolated, as if some patches were put on the worn surfaces. Those oxide films still could not resist the bare metal pairs directly contacting and cohering. With load increasing, the friction heat increased and the material in the junction region softened, which caused fatigue cracks which grew in the surface layer of the matrix (seen in Figure 8b,c). In this situation, the adhesive wear mechanism dominated over other wear mechanisms for 18Ni(300) maraging steel.

When sliding speed was greater than 50 m/s, the predominant composition of oxide film changed from FeO to Fe_3_O_4_ on worn surfaces (seen in Figure 7). It was found that the sliding speed had remarkable effects on the production rate and type of tribo-oxides. In these conditions, friction heat, *q*, was larger, and caused continuous, smooth, and thick (about 28 μm) tribo-oxide films to be formed on the worn surfaces (seen in Figure 8d). Those oxide films prevented direct metal–metal contact during the sliding process, resulting in the friction coefficient decreasing (seen in Figure 4). However, with load increasing, plastic deformation was more and more severe in the contact region. Consequently, fatigue cracks not only formed in the surface, but also in the subsurface of the matrix. Then oxide films were broken into small pieces, and delaminated from worn surfaces. The wear mechanism was a typical mild oxidative wear in this situation [11,12,18].

When the load was above 250 N at sliding speed 60 m/s, the wear condition was very harsh. Massive plastic deformation occurred in the subsurface layer of the matrix. Then, lots of inner cracks were initiated. Those cracks extended to the surface under the effect of plastic shear stress, and oxygen penetrated into the matrix through the cracks. Consequently, multi-layer tribo-oxide films were formed in matrix subsurface layers (seen in Figure 8f). In this situation, friction heat, *q*, was enormous and enough to cause the materials of rubbing pairs to melt in the junction regions. Then, the melted materials were extruded from the contact interface and spontaneously crystallized into spherical debris or formed an abrasive embedding in worn surfaces. In this situation, the wear mechanism was severe oxidative or extrusion wear, rather than a mild oxidative wear.

## 4. Conclusions

High-speed dry sliding tribo-tests of 18Ni(300) maraging steel have been studied. The results suggest the following conclusions:

(1) During the high-speed dry sliding process, three wear mechanisms of 18Ni(300) maraging steel are revealed. Under low load and sliding speed conditions (e.g., 100 N, 300 m/s), the adhesive wear mechanism is dominant. When sliding speed is greater than 50 m/s, smooth and thick tribo-oxide films form on the worn surface. In this case, the wear mechanism is a typical mild oxidational wear. When the load is above 250 N with sliding speed 60 m/s, the wear mechanism is severe oxidative or extrusion wear.
(2) The friction coefficient decreases with increasing sliding speed and load. One reason is attributed to materials being softened by frictional heat in the contact region, which leads to the decrease of frictional resistance; the other reason is that smooth and consecutive oxide films form on worn surfaces during rubbing, which may prevent adhesion of bare metal–metal and yield a lower friction.
(3) Wear rate increases with increasing sliding speed and load. However, the wear rate slowly increases under a low load, then rapidly increases when the load is more than a certain value. The abrupt change of wear rate reveals the transformation of the wear mechanism.
(4) Contact temperature, resulting from frictional heating, has an important influence on wear behavior and the formation of tribo-oxides. It is also a function of the product of load and speed and rises with the increasing of the product. The contact temperature is about 330 °C at 19.5 MPa·m/s (30 m/s, 100 N), and ferrous oxides (FeO) are formed on worn surfaces. When temperature is over 59 °C at 117 MPa·m/s (60 m/s, 300 N), the predominant composition of oxide film is changed from FeO to Fe_3_O_4_ on worn surfaces. The generation sequence of iron oxides is Fe→FeO→Fe_3_O_4_ with increasing of load and speed.

## Figures and Tables

**Table 1:** Chemical compositions of pin and disk specimens.

Sample	Steel	Elements (wt%)										
-	-	C	Si	Mn	Co	Ni	Mo	Ti	P	S	Cr	Fe
Pin	18Ni(300)	≤0.03	≤0.01	≤0.1	9.0	18.5	4.8	0.60	≤0.01	≤0.01	-	bal
Disk	100Gr6	1.05	0.35	0.35	-	≤0.3	≤0.1	-	≤0.02	≤0.02	1.5	bal
